# Unusually Large Submucosal Mandibular Lipoma of Buccal Vestibule: a Case Report and Review of Literature

**DOI:** 10.30476/DENTJODS.2021.87585.1268

**Published:** 2022-03

**Authors:** Karthik Ramakrishnan, Indu Palanivel, Vivek Narayanan, Munira Banu

**Affiliations:** 1 Dept. of Oral & Maxillofacial Surgery, SRM Kattankulathur Dental College & Hospital, Tamil Nadu, India

**Keywords:** Lipoma, Oral Lipoma, Mandibular, Buccal Vestibule, Soft tissue

## Abstract

Despite being one of the most common mesenchymal benign tumors in the body, lipomas in the oral cavity constitute only 1-4% of all benign neoplasm. Buccal mucosa is the most common anatomic site within the oral cavity which is followed by tongue, lips, floor of the mouth, palate, vestibule, retromolar area and gingiva. The average tumor size ranges from 10 to 30mm. We present a rare case of unusually large sub-mucosal lipoma in lower buccal vestibule measuring about 52×29×22mm at its greatest dimension.

## Introduction

Lipomas are called as ubiquitous or universal tumor. Lipomas, the common mesenchymal neoplasm of human body constitute mature adipocytes. These adipocytes were usually encircled with a fibrous capsule. Despite 15-20% of lipoma involved in head and neck, intraoral lipoma illustrates only 1-4% [ 1 ]. 

Oral lipoma was first described in 1848 as “Yellow Epulis” in a review of alveolar mass by Roux [ 2 ]. Lipomas are common in age groups between 40 and 50 years with no gender predilection. Common site for oral lipoma is buccal mucosa, followed by tongue, lips, floor of the mouth, vestibule, palate, gingiva, and retromolar region. Clinically, oral lipoma appears as a well circumscribed, soft, non-tender, slow growing tumor with a pedunculated or sessile base. The average size of these tumors ranges from 10 to 30mm [ 3 ]. The differential diagnosis is usually made with the traumatic fibroma, neurofibroma, granular cell and other salivary gland tumors.

Lipoma in the oral cavity is rare and in the mandibular vestibule, it is relatively unusual. Although a few cases were described, they were usually less than 30mm in size and in most of the cases, measurements with regard to the size were lacking. Here we document a case of unusually largest submucosal lipoma in the infrequent region of buccal vestibule of mandible with review of literature.

## Case Presentation

A 53-year-old man reported to authors department with a chief complaint of painless swelling in the left mandibular vestibule since 2 years. 

**Figure 1:**
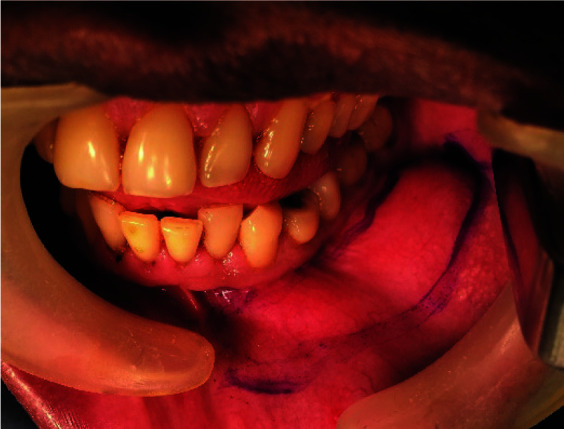
The extension of soft tissue swelling in the lower buccal vestibule from the midline till the molar region

The swelling size was gradually increased and attained the present size. His medical, dental, family and personal history was noncontributory. Clinically, the swelling was fluctuant, sessile, lobulated mass extending from midline to molar region in the buccal vestibule with smooth margins (Figure 1). 

The oral mucosa over the swelling had a yellow hue. It was non-tender and solitary. No neurological defects were demonstrated. Extraoral examination revealed no regional lymphadenopathy. Provisional diagnosis of lipoma was made. Differentiated diagnosis of salivary gland neoplasm and neurofibroma were considered.

**Figure 2:**
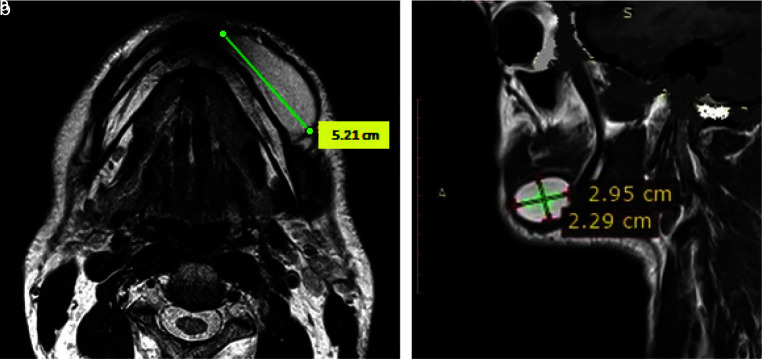
Pre-operative Magnetic Resonance Imaging showing a well-defined hyper dense lesion in the mandibular vestibule. **a:** Axial view, **b:** Sagittal view

MRI revealed horizontally ovoid T_1_/T_2_ hyper-intense lesion measuring 52×29×22mm at its greatest diameter in left lower gingivo-buccal sulcus with their hypointense septa (Figure 2). Adjacent mandibular cortex appeared normal in signal intensity and no soft tissue infiltration was found. No evidence of significant vascularity was noted. 

**Figure 3:**
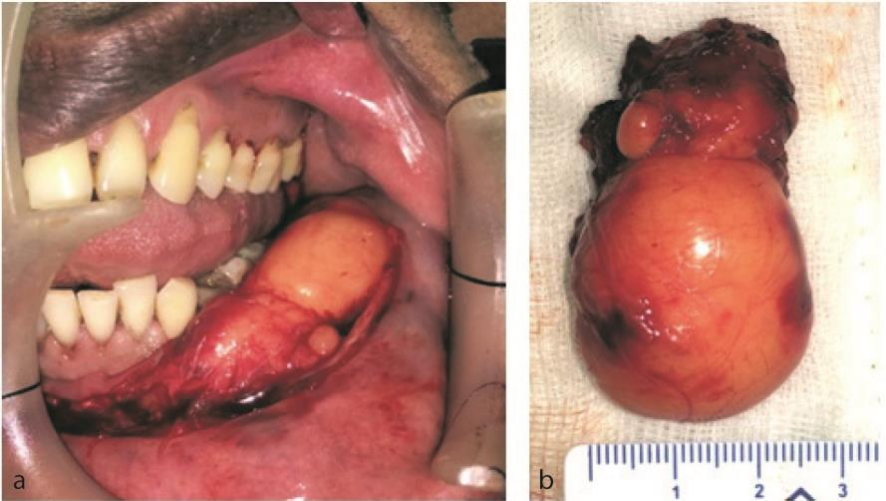
Excision of the tumor. **a:** Intra-oral view of lipoma, **b:** Excised specimen

**Figure 4:**
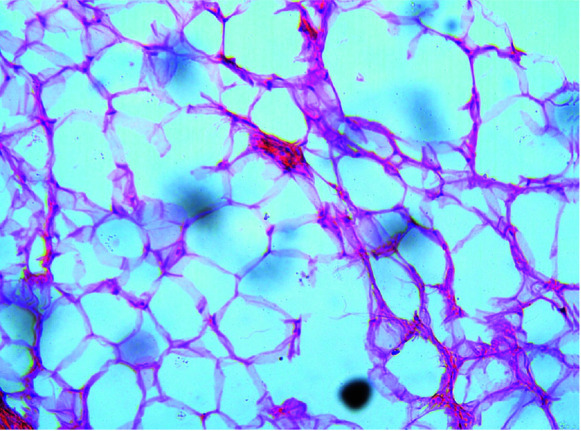
10x view of H& E stained section shows mature fat cells with slight variation in cellular size and shape with well vascularized vascular channels

Surgical excision was performed under general anesthesia. Yellowish, lobulated mass enclosed by a fibrous capsule was removed en bloc (Figure 3). Histological examination showed mature fat cells in varying size and shape. The adipocytes showed centrally positioned lipid vacuole and peripherally placed nuclei. Vascular channels are compressed by distended adipocytes (Figure 4). The final diagnosis of classical simple lipoma was made. The post-operative course was uneventful with no complications and there has been no recurrence until 18 months follow-up.

## Discussion

Lipomas are mesenchymal tumors of adipose tissue. Lipomas are common in age groups between 40 and 50 years with no gender predilection. Although very uncommon, oral lipomas have been reported even in children [ 4 ]. Although the precise pathophysiology and etiology are not clear, two main theories were proposed in the literature (i) Hypertrophy theory and (ii) Metaplasia theory. Hypertrophy theory connects obesity and inadequate adipose tissue growth with oral lipoma formation. It lacks explaining the rationale why lesion occurs in areas with no pre-existing adipose tissue. Metaplasia theory states that the lipomatous development occurs due to aberrant differentiation of mesenchymal cells in lipoblasts [ 5 ]. Factors like endocrine disorders, inflammation, hypercholesterolemia and obesity, radiation, chronic irritation, spontaneous development, metaplasia of muscle cells and fatty degeneration, trauma as well as chromosomal abnormalities have also been considered [ 6 ].

Lipomas are seen commonly in a region with excessive fat. Midface fat is divided into superficial cheek fat and deep cheek fat compartments. Buccal mucosa has abundant fatty tissue corresponding to 30.5% to 45.7% hence it is considered as the most frequent site. Lipoma in labial/buccal vestibule and gingiva were considered as the rare sites because it has negligible fat tissue or retroperitoneal space [ 1 ]. 

Fregnani *et al*. [ 1 ] reported a case series of intraoral lipoma. Of 46 cases, only 4 cases were found to be in the vestibule. Mary A Furlong *et al*. [ 7 ] studied 125 cases of oral lipomas and reported only 2 cases in the vestibule. Esther Manor *et al*. [ 8 ] analyzed 58 cases and reported only 5 cases in the vestibule. Taira *et al*. [ 3 ] studied 207 cases from 1987 to 2004 and reported only 4.8% cases in vestibule. The review of literature performed by Egido-Moreno *et al*. [ 9 ] in 2016, reported 95 cases from 2004-2014 and only 6 cases were described in the vestibule.

Sugimoto *et al*. [ 10 ] categorized the sites of development of oral lipoma cases, which was reported between 1935 and 1987 in Japan and found 5.9% occurrence in the vestibule. Studart Soares *et al*. [ 11 ] reviewed 450 intra-oral lipoma (from 1966 to 2009), and reported vestibule to be the second most common site but they did not include tongue and lips in their review. 

Oral lipomas usually appear as a soft, mobile, globular tumor with yellow colored mass. In our case, the oral mucosa appeared normal as the lesion was sub-mucosal. However, there was a yellow hue noted on the posterior aspect of the oral mucosa. 

The most frequent size of the tumor varies from 10 to 30mm. Oral lipomas larger than 30mm in size were proportionately infrequent [ 3 ]. Tumors of size more than 50mm reported in the literature are very rare. Smith [ 12 ] in 1937 has reported a huge lipoma of tongue measuring 110×90×70mm. Dattilo *et al*. [ 13 ] reported giant lipoma of tongue which measured 100×90×60mm. Marry A. Furlong *et al*. [ 7 ] reported 80mm large lipoma in the buccal mucosa. They also found lipomas were largest in the palate of their case series with a mean size of 60mm. Despite the limitation of growth, oral lipomas can even reach to a great dimension which interferes with mastication and speech thereby reinforcing the necessity for excision. 

Fregnoni *et al*. [ 1 ] studied the proliferative activity of lipomas and found differences in proliferating cell nuclear antigen (PCNA) and Ki-67 expression in the different histological groups They also mentioned the increased PCNA expression can be a contributing factor for faster growing tumors.

Histologically, oral lipomas were categorized based on matrix and preparation of tumor cells as classic or simple lipoma, fibro-lipoma, intramuscular lipoma, spindle lipoma, angiolipoma, pleomorphic lipoma, chondrolipoma, myxolipoma, osteolipoma, sialolipoma, angio-myxolipoma, infilterating lipoma, perineural lipoma, intra neural and atypical lipoma. The common histological subtype of oral lipoma is classic or simple lipoma [ 7 ].

Intra-muscular lipoma is characterized by a well-differentiated adipocytes interspersed by skeletal muscle fibers. Mature adipocytes are interspersed by dense and thick bundles of fibrous connective tissue, characterizing a fibrolipoma [ 14 ].

Histologically, spindle cell lipoma is characterized by bland spindle cells intermixed with ropey collagen bundles and mature adipose tissue in varying proportions. In addition, the presence of scattered mast cells and the strong expression of CD34 by the spindle cell component are characteristic. Pleomorphic lipoma is characterized by the presence of hyperchromatic cells and multinucleated floret-type giant cells, in addition to the spindle cell component [ 15 ].

The mean duration of tumor prior to excision in the literature is in the range of 2.5- to 3.2 years. The slow growing and painless nature of the tumors may be the attributing factors for the patients to seek medical attention at a later stage.

Treatment of choice for oral lipoma is the surgical excision. No recurrence was described; however, it may exist in infiltrating lipomas because of inadequate excision with non-capsulated lesion. However, advantages of suction assisted lipectomy for large lipomas have been reported [ 11 ]. Very few cases of a malignant transformation have been reported [ 11 ]. Differential diagnosis includes fibroma, dermoid cyst, sarcoma, malignant lymphoma, minor salivary gland tumors, neuroma and hemangioma. MRI seems useful peculiarly when the tumor is large and it exists in a deep region. The management of lipoma in consideration with all histological variants is surgical excision. Informed written consent was obtained from the patient for publication purpose.

## Conclusion

The significance of this report is its rare entity with regard to size and site. To authors’ knowledge, this is the radiologically well-documented largest oral lipoma in the buccal vestibule ever reported in English literature.

## Conflict of Interest

No conflict of interest is declared.
